# Yield & accuracy of Gastric Lavage in non-expectorating adults with Suspected Pulmonary Tuberculosis

**DOI:** 10.12669/pjms.39.5.6972

**Published:** 2023

**Authors:** Lubna Ghafoor, Faisal Faiyaz Zuberi, Ghulam Muqtada Khan, Maria Ismail

**Affiliations:** 1 Lubna Ghafoor, MBBS, MD Pulmonology Trainee Chest Unit-I, Ojha Institute of Chest Diseases, Dow University of Health Sciences, Karachi, Pakistan; 2 Faisal Faiyaz Zuberi, MBBS, FCPS (Med), FCPS (Pulm), FCCP Chest Unit-I, Ojha Institute of Chest Diseases, Dow University of Health Sciences, Karachi, Pakistan; 3 Ghulam Muqtada Khan, MBBS, FCPS (Pulm) Senior Registrar Pulmonology, Liaquat University of Medical & Health Sciences, Jamshoro, Pakistan; 4 Maria Ismail, MBBS, FCPS (Pulm) Consultant Pulmonologist, Karachi, Pakistan

**Keywords:** Pulmonary Tuberculosis, Mycobacterium Tuberculosis, gastric lavage, Anti-TB drugs, GXP, yield

## Abstract

**Objective:**

To determine the yield of Gastric lavage (GL) in non-expectorating adults with suspected Pulmonary Tuberculosis (PTB) and accuracy of GL-AFB smear with GL-GeneXpert (GXP) by taking AFB culture as gold standard.

**Methods:**

Cross-sectional study on suspected PTB patients was done at Ojha Institute of Chest Diseases during period 16^th^ July 2020 till 15^th^ January 2021. Adult patients of either gender suspected to have PTB and not expectorating were included. GL was performed and sent for AFB smear, GXP and AFB culture. Odds ratio, sensitivity and specificity were calculated.

**Results:**

After informed written consent, 206 patients, mean age was 38.17 ±17.30 years were inducted, including 89 (43.2%) males and 117 (56.8%) females. Gene Xpert, AFB smear & AFB culture were positive in 83(40%), 50 (24%) & 72 (35%) respectively in GL samples. Odds of PTB were 3.95 times higher among patients with ≤1 month of duration of symptoms (aOR 3.95, 95% CI 1.82-8.57, p-value 0.001), 6.24 times higher among patients with weight loss (aOR 6.24, 95% CI 3.03-12.84, p-value <0.001), and 4.22 times higher among patients with cavitation (aOR 4.22, 95% CI 1.99-8.93, p-value <0.001). GL-AFB smear showed sensitivity 63.89%, specificity 97.01%, positive predicted value 92%, negative predicted value 83.3%, and overall diagnostic accuracy 85.4%. Whereas GL-GXP showed sensitivity 94.4%, specificity 88.81%, positive predicted value 81.93%, negative predicted value 96.75%, and overall diagnostic accuracy 90.78%.

**Conclusion:**

Yield of GL significant to detect PTB in suspected cases who are not expectorating. GL-GXP diagnostic accuracy and sensitivity is higher than GL-AFB smear.

## INTRODUCTION

Pulmonary Tuberculosis (PTB), is one of the well-known chronic granulomatous diseases, affecting a large number of populations worldwide. Mycobacterium tuberculosis; an acid fast, alcohol fast, aerobic airborne bacterium is the reason behind such inevitable spreading of PTB.^1^ PTB affected the one third population of world in which 33% population have active PTB.^1^ World Health Organization (WHO) presented in their 2021 global tuberculosis report that 10.0 million new PTB patients were registered in 2020 throughout the world, among which majority were men. More precisely 58 percent cases were male as compared to 32 percent female and 10% children.^2^

It is unfortunate that Pakistan ranks fifth among countries severely affected with PTB and counts 60% of total new PTB cases. Pakistan is among the eight countries that constitute two third of the total PTB cases worldwide with total number of cases registered in the country mounting to 570,000 patients with prevalence of 5.7%.^2^ Presently, increasing frequency of PTB cases coupled with emergence of resistance against anti-TB drugs are the major hurdles in PTB management.^3^ These circumstances provoked the greatest interest and necessity for early diagnosis of PTB and anti-TB drugs sensitivity testing.^1^

For initial diagnosis and confirmation of PTB, Acid Fast Bacillus (AFB) test was used. However, half of the suspected active PTB cases are non-expectorating or demonstrated negative sputum smear.^4^ For diagnosis of PTB in such individuals alternative methods are used including Gastric lavage (GL), Bronchoalveolar Lavage (BAL) and Sputum Induction (SI).^5^ However, these substitute methods have merits and demerits of their own. For instance, BAL is an invasive procedure which is not possible in all regions of developing countries due to limited resources and other constraints.^6^ Likewise Sputum Induction showed satisfactory diagnostic results of PTB detection, but it required isolated room along with negative pressure which is also not available in all regions of developing countries.^7^

Among these alternate techniques, Gastric Lavage is convenient and preferred procedure for PTB detection in non-expectorating adults.^4^ Number of studies done on GL for early detection and confirmation of PTB come up with good results, favoring GL as feasible solution to overcome this problem of non-expectoration.^8^

## METHODS

A prospective cross-sectional study conducted during the period of 06 months from 16^th^ July 2020 till 15^th^ January 2021 at OJHA institute of chest disease, Dow University Hospital OJHA Campus, Karachi.

### Ethical Approval:

Study has IRB approval from DUHS Letter # IRB-589/DUHS/Approval/2020/702 dated 15-07-2020.

Total 206 non-expectorating patients of either gender aged between 18-65 years were enrolled using non-Probability purposive sampling technique after informed consent. Selected patients for the study did not include any patient who were already confirmed cases of PTB and patients taking ATT. Patients with history of known GI disorder like hematemesis, vomiting, nausea, CA stomach, peptic ulcer, esophageal varices, band ligation, sclerotherapy, clotting disorder were also excluded.

Patient’s medical history, physical examination and clinical investigation including x-ray and gastric lavage was done. Gastric lavage was performed for each non-expectorating patient for confirmation of PTB. For GL, informed consent from patients was obtained and they were advised fasting for at least 6-8 hours before procedure. An appropriately sized nasogastric tube (NG) was inserted via trans-esophageal into stomach. 30-50 ml of normal saline instilled in the stomach through nasogastric tube, followed by collection of instillations in sterile container, containing 100 mg of Sodium bicarbonate for neutralization. The collected sample was forwarded to the laboratory for AFB smear, GXP, and AFB culture. SPSS version 22 was utilized for analysis of collected data.

Factors including age, gender, duration of symptoms, presenting complains and radiological findings was stratified with PTB and diagnostic accuracy of AFB smear and GXP was calculated by applying Chi-square test, taking *p*-value ≤0.05 as significant.

## RESULTS

During the study period of six months, total of 206 patients were evaluated and their data was analyzed. Gender distribution showed that 89 were males and 117 females. Average age of patients was 38.17 ±17.30 years. The mean duration of symptoms was 1.61 ±1.58 months. Majority of the patients were presented with ≤1 month of duration of symptom (Table-I). Among the presenting complaints of the patients, that fever was reported by majority of the patients, i.e., 151, followed by dry cough 129, weight loss 106 while shortness of breath was reported by 71 patients. (Table-II). The radiological findings of the patients showed that nodular opacities were reported by 96 consolidations by 89, cavitation by 57, and miliary shadows by 17 patients. (Table-I) The findings of AFB smear showed positive cases in 50 (24%) patients while the findings of gene expert showed positive cases in 83 (40%) patients. Moreover, AFB culture positive cases in 72 (35%) of the patients. The frequency of PTB was observed in 72 (35%) patients.

**Table-I:** Demographic Characteristic, Presenting Complaints and Radiological Findings.

Demographic Characteristics	N	%
Age, years	38.17 ±17.30*	
≤35	111	53.9
>35	95	46.1
***Gender***		
Male	89	43.2
Female	117	56.8
Duration of symptoms, months	1.61 ±1.58*	
≤1	136	66
>1	70	34

***Presenting Complaints***	***n***	***%***

Dry Cough	129	62.6
Fever	151	73.3
Shortness of breath	71	34.5
Weight loss	106	51.5
Radiological Findings	n	%
Cavitation	57	27.7
Consolidation	89	43.2
Nodular Opacities	96	46.6
Miliary Shadows	16	7.8

* mean ±SD, n: number.

**Table-II:** Diagnostic accuracy of Gene expert taking AFB culture as gold standard (n=206).

GXP	AFB Culture		

	Positive	Negative	Total
Positive	68	15	83
Negative	4	119	123
Total	72	134	206

Sensitivity: 94.44% (95% CI: 86.38%-98.47%), Specificity: 88.81% (95% CI: 82.21%-93.60%), Positive Predicted Value (PPV): 81.93% (95% CI: 73.72%-87.99%), Negative Predicted Value (NPV): 96.75% (95% CI: 91.97%-98.72%), Overall Diagnostic Accuracy: 90.78% (95% CI: 85.97%-94.36%).

The comparison of pulmonary tuberculosis with demographic characteristics shows a significant association with duration of symptoms (p=.003). However, age (p=.069) and gender (p=.413) were statistically non-significant. Likewise, a significant association of PTB was observed with fever (p<.001) and weight loss (p<.001). Whereas dry cough (p=.743) and shortness of breath (p=.134) were found to be insignificant. Moreover, comparison of pulmonary tuberculosis with radiological findings showed a significant association of pulmonary tuberculosis was observed with cavitation (p<.001) only.

The univariate analysis showed the odds of PTB were 2.64 times higher among patients with ≤1 month of duration of symptoms when compared to patients with >1 month (OR 2.64, 95% CI 1.36-5.14, p=0.004). However, it was 6.59 times higher among patients with weight loss as compared to the patients without weight loss presenting complaint (OR 6.59, 95% CI 3.38-12.87, p<.001). It was 4.82 times higher among patients with cavitation (OR 4.82, 95% CI 2.51-9.22, p<.001). Similar findings were also observed in multivariable analysis. After adjustment of all other covariates, the odds of PTB were 3.95 times higher among patients with ≤1 month of duration of symptoms as compared to >1 month of duration (aOR 3.95, 95% CI 1.82-8.57, p=.001). It was 6.24 times higher among patients with weight loss as compared to the patients without weight loss (aOR 6.24, 95% CI 3.03-12.84, p<.001) and 4.22 times higher among patients with cavitation (aOR 4.22, 95% CI 1.99-8.93, p<.001).

Diagnostic accuracy of AFB smear taking AFB culture showed that sensitivity was found to be 63.89%, specificity 97.01%, positive predicted value 92%, negative predicted value 83.3%, and overall diagnostic accuracy 85.4%. Whereas the diagnostic accuracy of gene expert showed that sensitivity was found to be 94.4%, specificity 88.81%, positive predicted value 81.93%, negative predicted value 96.75%, and overall diagnostic accuracy 90.78% (Table-II & III).

**Table-III:** Diagnostic accuracy of AFB smear taking AFB culture as gold standard (n=206)

AFB smear	AFB Culture		

	Positive	Negative	Total
Positive	46	4	50
Negative	26	130	156
Total	72	134	206

Sensitivity: 63.89% (95% CI: 51.71%-74.88%), Specificity: 97.01% (95% CI: 92.53%-99.18%), Positive Predicted Value (PPV): 92% (95% CI: 81.18%-96.84%), Negative Predicted Value (NPV): 83.33% (95% CI: 78.60%-87.19%), Overall Diagnostic Accuracy: 85.44% (95% CI: 79.87%-89.95%)

Among 50 patients with AFB smear positive in gastric lavage, 49 (98.0%) had gene expert positive findings whereas 1 (2.0%) were gene expert negative. Whereas, among these 49 gene expert positive cases, AFB culture showed positivity in 46 (93.9%) patients. Among 83 patients with gene expert positive, AFB smear was found positive in 49 (59.0%) patients while negative in 34 (40.9%) patients. Furthermore, AFB culture was found positive in 68 (81.9%) and negative in 15 (18.1%) patients. There was only one patient with AFB smear positive but had negative GXP. Moreover, this patient had NTM Positive in AFB Culture. Among 72 patients with AFB Culture positive PTB, 51 (70.8%) were drug sensitive while 21 (29.2%) were drug resistant. Specification given in Table-IV.

**Table-IV:** Specification of drug resistance (n=21).

Drug Resistance	n (%)
INH	3 (14.2)
PZA	6 (28.5)
Levofloxacin	5 (23.8)
Streptomycin	1 (4.7)
Fluoroquinolone	2 (9.5)
PZA, Streptomycin, Levofloxacin	1 (4.7)
INH, Rifampicin, Levofloxacin	1 (4.7)
INH, Rifampicin	1 (4.7)
INH, Rifampicin, PZA, Streptomycin	1 (4.7)

INH: Isoniazid, PZA: Pyrazinamide.

## DISCUSSION

It has been observed that very little work is done in Pakistan on effectiveness of gastric lavage in diagnosing the pulmonary tuberculosis in non-expectorating adults with suspected pulmonary tuberculosis. in non-expectorating adults with suspected pulmonary tuberculosis.^1^,^3^ In current study, 206 suspected pulmonary tuberculosis patients were evaluated with difficulties in expectorating sputum. Gastric content was collected in sterile container by following standard protocol and tested in laboratory for AFB smear, GXP and AFB culture.

Diagnostic accuracy of GXP of gastric lavage showed that sensitivity to be 94.4%, which is higher when compared with sensitivity of AFB smear of GL which is 63.89%.^9^-^12^ A similar finding observed in study by Aslam W et al also reported the higher GXP sensitivity (82.8%) as compared to AFB smear (61.0%) of gastric lavage.^8^ A study by Baghaei P et al from Iran also reports the similar sensitivity of AFB smear of gastric lavage (66.07%).^13^ A systematic review on twenty seven published papers collected throughout the world was conducted by Maynard-Smith L et al also reported the 95% sensitivity of GXP of gastric lavage in smear positive and 65% in smear negative patients.^14^ Sensitivity of GXP of gastric lavage was high and comparable to gold standard and can replace the culture for rapid diagnosis and initiating treatment of pulmonary tuberculosis.^8^,^13^,^15^ GXP of gastric lavage can also be used in case of unavailability of culture.^9^,^16^

In this study, pulmonary tuberculosis was diagnosed in 72 (35%) patients based on positive AFB culture of gastric lavage, which was used as gold standard, whereas AFB smear of gastric lavage was positive in 50 (24%) patients and GXP of gastric lavage in 83 (40%) patients. Similarly, other Pakistani study by Aslam W et al also reported the higher detection of PTB 34.8% with gastric specimen.^8^ Overall study also reports higher positivity with GXP 30.3% followed by culture with 24.9% and smear with 23.6%. Similarly, another Pakistani study by Mahat R et al also reported the higher positivity of gastric lavage with GXP 38.9%.^17^ Another study of Brown M et al from United Kingdom also reported the pulmonary tuberculosis in 43% patients on evaluation of gastric lavage. Researcher also reports higher positivity of gastric lavage with gene culture 30% followed by smear with 13%.^16^,^18^,^19^

Rate of diagnosis was high in this study due to selection of suspected patients of pulmonary tuberculosis that were reported positive to different clinical symptoms and radiological findings. This study has advantages over previous Pakistani study^8^ as it was cross-sectional study and current data of suspected cases of pulmonary tuberculosis was collected that decreased the risk of incomplete data. Previous study was retrospective with limitation of incomplete data regarding medical history, clinical data, treatment and its outcomes, whereas this study overcomes these problems and collects all data regarding clinical symptoms and radiological findings that helps in identifying suspected cases of pulmonary tuberculosis and finally gold standard helps to diagnose the pulmonary tuberculosis.

In this study, out of seventy-two patients diagnosed with PTB, 51 were drug sensitive while 21 were drug resistant. This is observed that by doing so we not only diagnosed drug sensitive TB but also can timely diagnosed drug resistant TB. Hence by timely and appropriately managing patients we can reduce morbidity and mortality attributed to this disease.

### Limitations:

Being a single center study, the results could not be generalized. Multicenter study with large sample size is advised.

## CONCLUSION

Gastric lavage sample is helpful in detecting pulmonary tuberculosis in suspected cases who are unable to expectorate. GXP of gastric lavage sample not only is more appropriate and rapid in diagnosis of pulmonary tuberculosis as compared to AFB smear of gastric lavage but also diagnostic accuracy and sensitivity of gastric lavage GXP is higher than that of AFB smear.

### Authors Contribution:

**LH:** Study conception, data collection.

**FFZ:** Final approval and responsible of the study.

**GMK:** Draft manuscript.

**MI:** Statistical analysis.
